# The role of short-chain fatty acid metabolism in the pathogenesis, diagnosis and treatment of cancer

**DOI:** 10.3389/fonc.2024.1451045

**Published:** 2024-10-07

**Authors:** Maolin Hou, Qing-Qing Yu, Le Yang, Haibo Zhao, Pei Jiang, Lei Qin, Qiujie Zhang

**Affiliations:** ^1^ Department of Internal Medicine, Siziwangqi People’s Hospital, Wulancabu, China; ^2^ Translational Pharmaceutical Laboratory, Jining NO.1 People’s Hospital, Jining, China; ^3^ Department of Gastrointestinal Surgery, Jining NO.1 People’s Hospital, Jining, China; ^4^ Department of Oncology, Jining No.1 People’s Hospital, Jining, China

**Keywords:** short-chain fatty acid, metabolism, cancer, pathogenesis, diagnosis, treatment

## Abstract

Short-chain fatty acids (SCFAs), which are saturated fatty acids consisting of six or fewer carbon atoms, have been found to be closely associated with the biological behavior of malignant tumors. This manuscript provides a comprehensive review on the role of SCFAs in regulating cell cycle, apoptosis, tumor angiogenesis, epithelial-mesenchymal transition, protein regulatory pathways, and histone regulation in promoting the development of malignant tumors. Furthermore, we discuss the potential therapeutic strategies targeting SCFAs for treating malignant tumors. This review offers a theoretical foundation for investigating the mechanisms by which SCFAs impact malignant tumors and provides insights into developing novel treatment targets.

## 1 Introduction

Short-chain fatty acids (SCFAs) are saturated fatty acids consisting of six or fewer carbon atoms. Due to their low molecular weight, they readily volatilize at room temperature and are also referred to as volatile fatty acids (VFAs) (1). The majority of SCFAs in the human body are metabolic byproducts resulting from the fermentation of dietary fiber by anaerobic bacteria or yeast in the colon. The types and quantities of SCFAs primarily rely on the composition of gut microbiota, digestion time, host microbial metabolism flux, and fiber content in the host’s diet. Acetate, propionate, butyrate, and isovalerate (lactate) are among the most prevalent SCFAs. Acetate and propionate are predominantly produced by Bacteroides while butyrate is synthesized by Firmicutes (2). SCFAs play a crucial role in maintaining energy supply, regulating motility, and safeguarding mucosal barrier integrity within the intestine (3).

**Figure 1:**
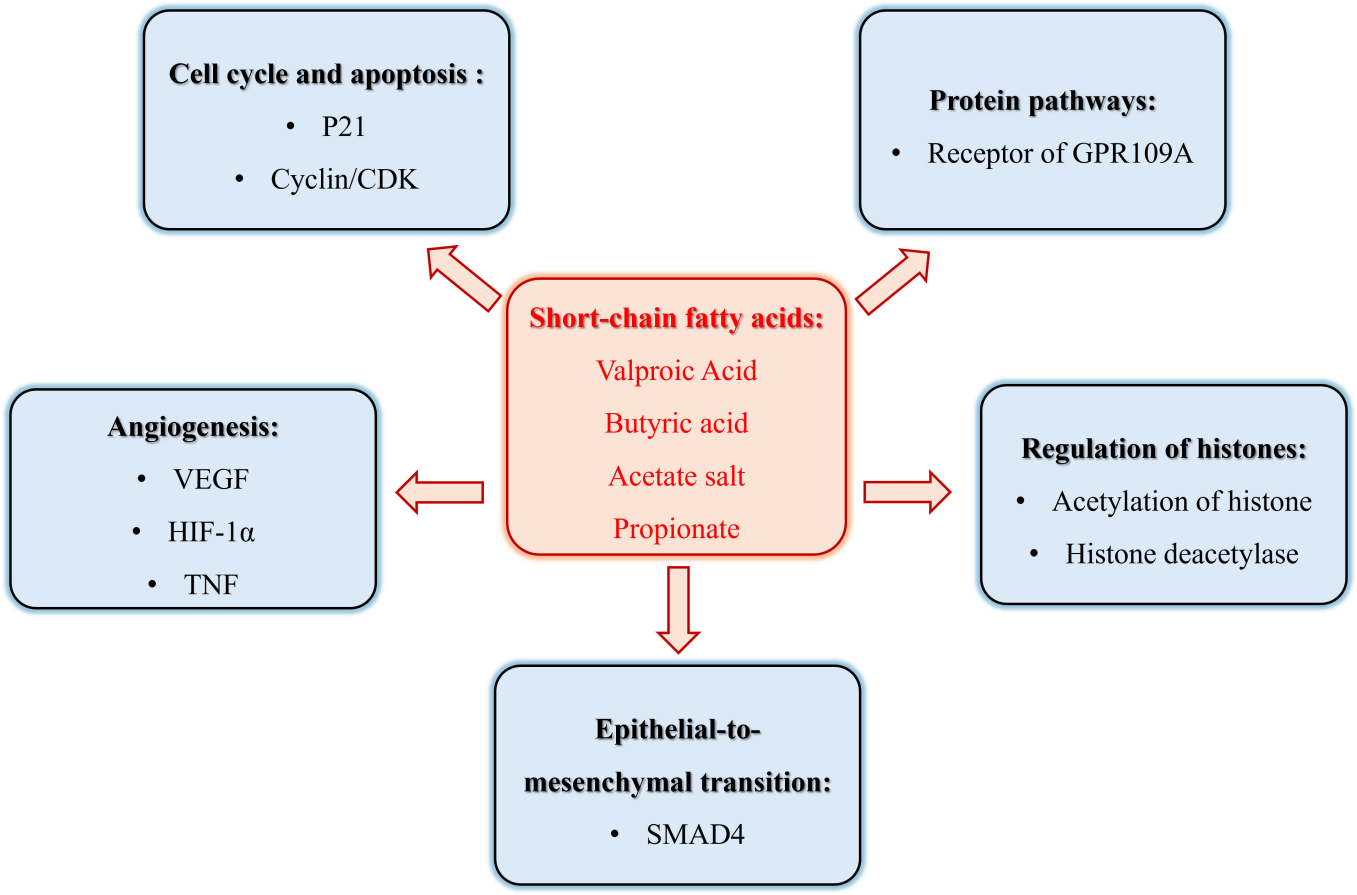
The role of short chain fatty acid metabolism in the pathogenesis of cancer.

In recent years, the role of SCFAs in tumor pathogenesis has garnered significant attention, with a particular focus on Valproic Acid (VPA), Butyric Acid, Acetate Salt, and Propionate, as shown in **Table 1**. These SCFAs have been extensively studied for their anti-tumorigenic properties and underlying mechanisms. However, despite the compelling evidence presented by these compounds, research exploring the effects of other SCFAs, such as Caproic Acid (4) and Succinate (5), on tumor growth and progression remains relatively scarce, particularly in terms of elucidating their specific mechanisms of action. Therefore, this review has unveiled the role of mainly SCFAs in malignant tumor biology, as in **Figure 1**.

**Table 1:** The mechanism of SCFAs depending on the cancer type.

Cancer type	SCFAs	Mechanism	Reference
Lung cancer	Propionate	Apoptosis and cell cycle arrest	(21)
Breast cancer	Butyric acid	Receptor of GPR109A	(36)
Colon cancer	Butyrate	Receptor of GPR109A	(33)
		Acetylation of histone	(42)
		Histone deacetylase	(40)
Hepatocarcinoma cells	VPA	Cell cycle arrest	(14)
Pancreatic cancer	VPA	Cell cycle arrest, apoptosis	(9)
Renal cell carcinoma	VPA	Epithelial–mesenchymal transition	(29)
Prostatic cancer	VPA	Epithelial–mesenchymal transition	(28)
Cervical cancer	VPA	Angiogenesis	(25)
Glioma	VPA	Cell cycle arrest (G1)	(10)
Glioblastoma	VPA	Acetylation of histone	(41)

## 2 The role of SCFAs in cell cycle and apoptosis of cancer

**Figure 2:**
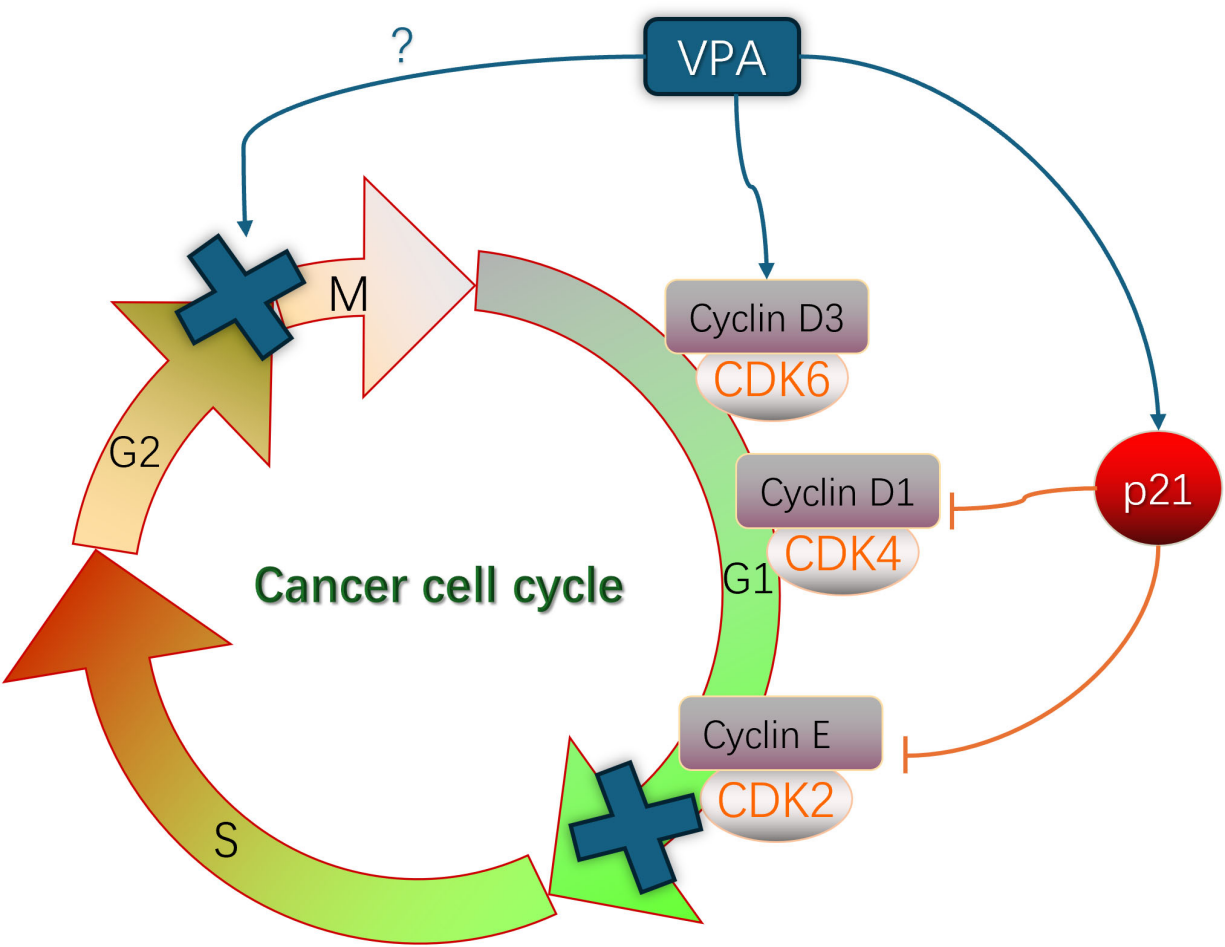
The role of VPA in the regulation to cancer cell cycle involved in the mechanism of cancer.

The hallmark of tumor cells is aberrant proliferation, and the cell cycle is tightly regulated through the modulation of proteins (Cyclin), Cyclin-dependent kinases (CDKs), and Cyclin-dependent kinase inhibitors (CKIs) to govern cellular growth and division. Cyclin assumes a pivotal role throughout the entirety of the cell cycle process, ensuring precise regulation at distinct stages of cell cycle progression while mediating checkpoint functions (6). These three regulatory factors primarily achieve their functions through Rb pathway and p53 pathway (7, 8). Research has demonstrated that SCFAs can induce cell cycle arrest and inhibit cell proliferation in tumor cells by modulating factors associated with the cell cycle. Valproic acid (VPA) induces the expression of p21 and topoisomerase II (α/β), where p21 acts as a conventional cell cycle inhibitor, restraining the activity of cycDl-CDK4 and cycE-CDK2, thereby leading to G1 phase arrest (**Figure 2**) (9). Studies conducted by Bacon C L (10) have revealed that exposure to VPA significantly upregulates Cyclin D3 expression during mid-G1 phase and translocates it into the nucleus in glioma cells. Cyclin D plays a pivotal role as a core component driving cellular division throughout the cell cycle, with Cyclin D1 typically expressed during early G1 phase and Cyclin D3 expressed during late G1 phase (**Figure 2**) (11), suggesting that elevated levels of Cyclin D3 expression and ectopic activation are crucial determinants for VPA-induced G1 phase arrest. Through the Warburg effect, differentiated tumor cells primarily rely on glycolysis to meet their energy demands. It has been discovered that Cyclin D3 can phosphorylate and deactivate PFK1 (phosphofructokinase 1) and PKM2 (pyruvate kinase m2), consequently inhibiting glycolysis during G1 phase while weakening energy supply to tumor cells (12, 13). The molecular mechanisms underlying short-chain fatty acid-induced cell cycle arrest may vary depending on the specific type of cells, as VPA treatment has also been observed to induce G2/M phase arrest in lung cancer cells (13), whereas liver cancer cells treated with VPA experience both G2/M phase arrest and G0/G1 phases arrest (**Figure 2**) (14). Similarly, butyrate strongly arrested the cell cycle at G2 phase and promoted apoptosis, leading to tumor cell death (15, 16).

Cellular apoptosis is considered to be a crucial mechanism for preventing tumorigenesis; however, one hallmark feature of tumor cells is their ability to suppress apoptosis (17). In tumor cells, the equilibrium between pro-apoptotic and anti-apoptotic proteins such as the Bcl-2 protein family and IAPs becomes disrupted, resulting in attenuated caspase activity. The study revealed that SCFAs can modulate apoptosis-related proteins and impact the cellular apoptosis process. There are two primary pathways for initiating cell apoptosis: intrinsic and extrinsic pathways, both of which activate the Caspase family of proteases. Caspase has the ability to hydrolyze over 400 types of proteins, thereby accelerating cell death (18). Butyrate triggers the endogenous apoptotic pathway by regulating Bcl-2 activity, a mitochondrial-mediated apoptotic regulatory factor, upregulating BH3-only transcriptional activators, releasing pro-apoptotic factors BAX and BAK to form oligomers, increasing mitochondrial outer membrane permeability, releasing cytochrome C and Smac/DIABLO apoptotic factors from mitochondria. These apoptotic factors facilitate activation of Caspase 9 followed by activation of Caspases 3 and 7 leading to cellular apoptosis (19). In most cases, all apoptotic signals converge on the final executor Caspase-3; thus detecting the content or activity level of cleaved Caspase-3 reflects the progression of cellular apoptosis (20). Kim K et al. (21) discovered that propionate treatment significantly decreased Survivin expression levels while increasing p21 protein expression levels in H1299 and H1703 cells; early-stage and late-stage apoptotic cells were more abundant in SP-treated groups compared to control groups; caspase 3/7 activity was also notably increased.

## 3 The role of SCFAs in angiogenesis of cancer

Tumor growth and metastasis rely on angiogenesis, with VEGF playing a crucial role in promoting vascular formation and tumor cell proliferation. Targeting pro-angiogenic genes is an effective therapeutic strategy for inhibiting tumor progression (22). The research findings indicate that short-chain fatty acids (SCFAs) possess potential as anti-angiogenic drugs due to their impact on the expression of vascular endothelial growth factor (VEGF). Butyrate salts can downregulate VEGF expression by inhibiting the binding affinity of Sp1, a transcription factor, to the promoter region of neuropilin-1 (NRP-1), thereby reducing its capacity for expression. NRP-1 acts as a co-receptor for VEGF and enhances the interaction between VEGF165 and EGFR-2, promoting chemotaxis and mitogenesis in endothelial cells induced by VEGF165, thus facilitating VEGF-mediated angiogenesis (23, 24). Valproic acid (VPA) exhibits time-dependent inhibition of HIF-1α, VEGF, and tumor necrosis factor (TNF) expression in cervical cancer cells through suppression of PI3K/Akt and ERK1/2 signaling pathways. Additionally, it suppresses endothelial cell migration and regulates tumor neovascularization (25).

## 4 The role of SCFAs in epithelial-to-mesenchymal transition of cancer

Epithelial-to-mesenchymal transition (EMT) is a biological process wherein epithelial cells undergo a specific program to acquire mesenchymal characteristics, playing a crucial role in conferring migration and invasion abilities upon malignant tumor cells derived from epithelium. EMT leads to the loss of certain epithelial cell traits, resulting in decreased expression levels of key epithelial genes such as E-cadherin, ZO-1, and occludin. Consequently, there is reduced contact with surrounding and stromal cells, diminished intercellular interactions, and acquisition of mesenchymal cell features. Concurrently, the expression levels of mesenchymal genes like N-cadherin, vimentin, and fibronectin increase during this process. This ultimately enhances cell migration and motility while promoting increased invasive capacity and detachment capability. These effects can be mediated through the classical Smad pathway (26, 27). VPA exhibits the ability to downregulate SMAD4 protein levels—a pivotal factor in TGF-β-induced EMT—and effectively inhibits metastatic potential in prostate cancer (28) as well as renal cancer cells (29).

## 5 The role of SCFAs in protein pathways of cancer

The G-protein coupled receptors (GPCRs), also known as seven-transmembrane receptors, constitute the largest family of cell surface receptors in eukaryotes and participate in numerous cellular signaling pathways. Several members of the GPCR family have been implicated in tumor initiation and progression, including SMO protein - a pivotal component of the Hedgehog signaling pathway that, when aberrantly activated, can contribute to various cancers such as basal cell carcinoma and rhabdomyosarcoma (30). The chemokine receptor CXCR4 is frequently overexpressed in tumors and is believed to play a crucial role in angiogenesis, tumor cell migration, invasion, and metastasis (31). Short-chain fatty acids (SCFAs) serve as natural ligands for GPCRs. Upon binding, they further activate signal cascades mediated by phospholipase C, mitogen-activated protein kinases (MAPKs), phospholipase A2, and nuclear factor κB (32). In colon cancer cells, SCFAs can inhibit cell proliferation through NF-КB, MAPKs ERK1/2 PI3K, and Wnt signaling pathways while inducing apoptosis and cell cycle arrest (33, 34). GPCRs expressed in breast cancer cell lines can elevate intracellular calcium concentration upon binding with SCFAs thereby activating the p38 MAPK pathway to suppress tumor cell proliferation (35). Moreover, GPCRs like GPR109A selectively bind to butyric acid which mediates its anticancer activity thus reducing invasive capabilities of breast cancer cells (36).

## 6 The role of SCFAs in histones regulation mechanism of cancer

Proteins are small, alkaline proteins found in the chromatin of eukaryotic cells that, together with DNA, form nucleosomes. Modifications such as methylation, acetylation, phosphorylation, and ubiquitination of histones can alter chromatin structure and play a pivotal role in epigenetic regulation (37, 38). Acetylation is one of the most prevalent modifications of histones and its extent is finely regulated by the metabolic state of organisms. Histone acetylation facilitates the dissociation of DNA from histone octamers, thereby loosening the structure of nucleosomes. This enables specific binding of various transcription factors and coactivators to DNA binding sites, activating gene transcription. Transcriptionally active regions exhibit a high density of acetylated core histones while inactive regions have a low density. Conversely, histone deacetylases (HDACs) exert opposing effects by compacting DNA and inhibiting transcription processes (39). Histone acetylation and deacetylation modifications serve as major regulators for gene expression control. Alterations in nucleosome structure are crucial for precise gene expression in eukaryotic cells. Targeting HDACs holds significant potential for anti-tumor activity; HDAC inhibitors induce chromatin remodeling through increased levels of histone acetylation, rectifying epigenetic errors, promoting anti-tumor activity, and enhancing expression levels of tumor suppressor genes to inhibit cancer cell proliferation (40).

The HDACIs encompass a variety of compounds with diverse structures, including short-chain fatty acids like butyrate salts, butyrate esters, and valproic acid. Treatment with VPA in glioblastoma cell lines resulted in enhanced acetylation of histone H4 (41). Similarly, treatment with butyrate in colon cancer cells led to increased acetylation of histone H3, indicating the inhibition of HDACs within these cells (42). HDACIs exert their effects by regulating the extent of DNA wrapping around histones. Histone deacetylases remove acetyl groups from histones, resulting in tightly wrapped DNA that is less accessible to transcription factors. Consequently, this leads to the suppression of protein expression associated with cell cycle arrest and apoptosis in damaged cells as well as a decrease in the expression of tumor suppressor genes and other anti-cancer genes, thereby promoting cancer development (40). Butyrate salts can impede CRC cell migration and invasion by blocking the activation of HDAC3, which subsequently reduces phosphorylation levels of Akt1 and erk1/2 leading to inhibited cell motility (43).

## 7 The role of SCFAs in treatment of cancer

Supplementation with exogenous bacteria that produce short-chain fatty acids has been demonstrated to augment the efficacy and sensitivity of chemotherapy, radiotherapy, or immunotherapy in the treatment of malignant tumors, while concurrently mitigating treatment-related toxicity. Notably, butyrate salts have been observed to deplete vancomycin-sensitive bacteria in lung cancer and potentiate the anti-tumor activity of radiation therapy (44). VPA synergistically enhances the cytotoxicity of temozolomide in high-grade glioma cell lines. The combination of valproic acid and arsenic trioxide induces G2/M phase arrest and promotes apoptosis cell death, effectively inhibiting lung cancer cell growth by modulating the cell cycle. *In vivo* studies have substantiated a synergistic anti-tumor effect (45).

SCFAs exhibit a dose-dependent inhibition on colony formation and proliferation of colorectal cancer cells, regulate the composition of colonic microbiota in colon cancer, and enhance the proportion of SCFA-producing bacteria. Faecalibaculum rodentium and its human homolog biformholdemanella are two microbial strains discovered to possess anti-colon tumor properties (46). They demonstrate the ability to produce SCFAs in both mouse and human experiments, control protein acetylation and tumor cell proliferation by suppressing calcium-regulated phosphatase secretion while activating NFATc3, as well as inhibit the growth of tumor cell lines or patient tumor samples *in vitro* (47). Therefore, inducing an increase in SCFA content within the intestinal tract may potentially serve as an adjuvant therapy for colorectal cancer.

Butyrate can attenuate oxidative stress on the gastric mucosa, upregulate the expression of GPR109A, decrease the levels of pro-inflammatory factors such as TNF-α and IL-1β, and play a crucial role in gastric mucosal repair. Studies have demonstrated that oral supplementation of butyric acid bacteria post-gastrectomy can enhance intestinal SCFA content through fermentation of various carbohydrates, leading to reduced expression of inflammatory cells and factors, improved immune function, decreased postoperative complications, and enhanced recovery in patients following gastric cancer surgery (48). Moreover, there is evidence supporting that acetate salts possess dose-dependent apoptotic effects on gastric cancer cells and mesothelioma cells with heightened sensitivity towards human tumor cells. Local application of acetate combined with chemotherapy may represent a viable treatment approach and novel therapeutic strategy for drug-resistant mesothelioma (49). Tri-butyrylglycerol (a classical derivative of short-chain fatty acids) inhibits the activity of gastric cancer cells in a dose-dependent manner. Appropriate supplementation may exert preventive effects against gastric cancer (50). Kim et al. (51) discovered through plasma level analysis that propionate levels were significantly elevated in patients with gastric cancer, suggesting its potential as a novel biomarker for evaluating disease progression. Furthermore, it has been found that acetate salts also possess the ability to downregulate estrogen receptors in breast cancer and exhibit certain clinical efficacy in treating ER-positive endocrine-resistant breast cancer patients (52).

It has been confirmed that SCFAs may play an important regulatory role in the immune system in a complex manner. Previous studies have found a negative correlation between pre-treatment serum concentrations of butyrate and propionate and overall survival and progression-free survival in patients with metastatic melanoma receiving CTLA-4 monoclonal antibody therapy. High levels of butyrate in the blood inhibit the accumulation of memory T cells and ICOS^+^ CD4^+^ T cells induced by CTLA-4 monoclonal antibody, as well as reduce the efficacy of CTLA-4 monoclonal antibody in three different tumor mouse models. Butyrate also inhibits the upregulation of CD80/CD86 on dendritic cells and ICOS on T cells induced by CTLA-4 monoclonal antibody, while increasing the proportion of Tregs (53). However, further research has revealed that pectin, a major soluble fiber extracted from plant cell walls, can alter butyrate levels in humanized tumor-bearing mice with gut microbiota derived from colorectal cancer patients. It suppresses tumor growth in humanized mouse models resistant to anti-PD-1 monoclonal antibodies due to their gut microbiota composition, suggesting its potential ability to reverse resistance to anti-PD-1 monoclonal antibodies in colon cancer patients (53). Combining acetate salts with PD-1 therapy significantly delays the growth of hepatocellular carcinoma compared to administering acetate salts alone (54). Therapeutic supplementation of short-chain fatty acids (SCFAs) or a high-fiber diet, which enhances endogenous SCFA production, inhibits osteoclast activity and prevents pathological fractures (55). *In vitro* studies by Luu et al. (56) demonstrated that SCFAs modulate the activity of reprogrammed CD8^+^ CTLs and CAR-T cells by inhibiting HDAC, leading to increased production of effector molecules such as CD25, IFN-γ, and TNF-α. This augmentation strengthens mTOR’s role as a central cellular metabolic sensor in CD8^+^ T cells. mTOR influences cytokine expression in T cells and is involved in immune suppression, DNA transcription regulation, cell growth, and apoptosis. Consequently, it enhances the anti-tumor activity of T cells and significantly amplifies the anti-tumor efficacy of antigen-specific CTLs targeting ROR1 in melanoma and pancreatic cancer models using genetically modified mice. These findings have positive implications for improving the therapeutic efficacy of tumor immunotherapy and hold promise for optimizing CAR-T cell therapy as well as other tumor therapies through modulation of bacterial species within the gut microbiome.

## 8 Conclusions

SCFAs, primary metabolic byproducts of dietary fiber fermentation mediated by anaerobic bacteria and yeasts within the intestinal milieu, exhibit profound biological functions in human physiology. This comprehensive review meticulously examines the intricate mechanisms of SCFAs in malignancy, encompassing their intricate interplay with cell cycle regulation, apoptosis, tumor angiogenesis, EMT, and histone modulation, while also elucidating the pivotal roles of SCFAs-associated metabolic pathways in cancer progression. In summary, SCFAs occupy a central position in the intricate web of malignancy initiation, progression, and treatment. Future endeavors aimed at elucidating the intricate metabolic mechanisms of SCFAs and their therapeutic potential in cancer will undoubtedly yield invaluable insights, paving the way for the development of innovative anti-tumor strategies. By harnessing the power of gut microbiota modulation and SCFAs production, we may uncover novel avenues for the prevention and treatment of malignancies, thereby revolutionizing the landscape of cancer medicine.

## References

[1] Fei Q Chang HN Shang L Choi JD Kim N Kang J. The effect of volatile fatty acids as a sole carbon source on lipid accumulation by Cryptococcus albidus for biodiesel production. Bioresour Technol. (2011) 102:2695–701. doi: 10.1016/j.biortech.2010.10.141 21134744

[2] Levy M Thaiss CA Elinav E. Metabolites: messengers between the microbiota and the immune system. Genes Dev. (2016) 30:1589–97. doi: 10.1101/gad.284091.116 PMC4973288 27474437

[3] Parada Venegas D de la Fuente MK Landskron G González MJ Quera R Dijkstra G. Short chain fatty acids (SCFAs)-mediated gut epithelial and immune regulation and its relevance for inflammatory bowel diseases. Front Immunol. (2019) 10:277. doi: 10.3389/fimmu.2019.00277 30915065 PMC6421268

[4] Narayanan A Baskaran SA Amalaradjou MA Venkitanarayanan K. Anticarcinogenic properties of medium chain fatty acids on human colorectal, skin and breast cancer cells *in vitro* . Int J Mol Sci. (2015) 16:5014–27. doi: 10.3390/ijms16035014 PMC4394462 25749477

[5] Wu JY Huang TW Hsieh YT Wang YF Yen CC Lee GL. Cancer-derived succinate promotes macrophage polarization and cancer metastasis via succinate receptor. Mol Cell. (2020) 77:213–227.e5. doi: 10.1016/j.molcel.2019.10.023 31735641

[6] Santamaria D Ortega S. Cyclins and CDKS in development and cancer: lessons from genetically modified mice. Front Biosci. (2006) 11:1164–88. doi: 10.2741/1871 16146805

[7] Dyson NJ. RB1: a prototype tumor suppressor and an enigma. Genes Dev. (2016) 30:1492–502. doi: 10.1101/gad.282145.116 PMC4949322 27401552

[8] Engeland K. Cell cycle arrest through indirect transcriptional repression by p53: I have a DREAM. Cell Death Differ. (2018) 25:114–32. doi: 10.1038/cdd.2017.172 PMC5729532 29125603

[9] Gilardini Montani MS Granato M Santoni C Del Porto P Merendino N D’Orazi G. Histone deacetylase inhibitors VPA and TSA induce apoptosis and autophagy in pancreatic cancer cells. Cell Oncol (Dordr). (2017) 40:167–80. doi: 10.1007/s13402-017-0314-z PMC13001582 28160167

[10] Bacon CL Gallagher HC Haughey JC Regan CM. Antiproliferative action of valproate is associated with aberrant expression and nuclear translocation of cyclin D3 during the C6 glioma G1 phase. J Neurochem. (2002) 83:12–9. doi: 10.1046/j.1471-4159.2002.01081.x 12358724

[11] Kato JY Sherr CJ. Inhibition of granulocyte differentiation by G1 cyclins D2 and D3 but not D1. Proc Natl Acad Sci USA. (1993) 90:11513–7. doi: 10.1073/pnas.90.24.11513 PMC48014 7505440

[12] Yalcin A Clem BF Simmons A Lane A Nelson K Clem AL. Nuclear targeting of 6-phosphofructo-2-kinase (PFKFB3) increases proliferation via cyclin-dependent kinases. J Biol Chem. (2009) 284:24223–32. doi: 10.1074/jbc.M109.016816 PMC2782016 19473963

[13] Racker E. Bioenergetics and the problem of tumor growth. Am Sci. (1972) 60:56–63. 4332766

[14] An HM Xue YF Shen YL Du Q Hu B. Sodium valproate induces cell senescence in human hepatocarcinoma cells. Molecules. (2013) 18:14935–47. doi: 10.3390/molecules181214935 PMC6270308 24304587

[15] Bultman SJ. The microbiome and its potential as a cancer preventive intervention. Semin Oncol. (2016) 43:97–106. doi: 10.1053/j.seminoncol.2015.09.001 26970128 PMC4789109

[16] Zeng H Hamlin SK Safratowich BD Cheng WH Johnson LK. Superior inhibitory efficacy of butyrate over propionate and acetate against human colon cancer cell proliferation via cell cycle arrest and apoptosis: linking dietary fiber to cancer prevention. Nutr Res. (2020) 83:63–72. doi: 10.1016/j.nutres.2020.08.009 33017771

[17] Wong RS. Apoptosis in cancer: from pathogenesis to treatment. J Exp Clin Cancer Res. (2011) 30:87. doi: 10.1186/1756-9966-30-87 21943236 PMC3197541

[18] Schafer ZT Kornbluth S. The apoptosome: physiological, developmental, and pathological modes of regulation. Dev Cell. (2006) 10:549–61. doi: 10.1016/j.devcel.2006.04.008 16678772

[19] Zhang K Ji X Song Z Wu F Qu Y Jin X. Butyrate inhibits gastric cancer cells by inducing mitochondriamediated apoptosis. Comb Chem High Throughput Screen. (2023) 26:630–8. doi: 10.2174/1386207325666220720114642 35864794

[20] Liang Y Yan C Schor NF. Apoptosis in the absence of caspase 3. Oncogene. (2001) 20:6570–8. doi: 10.1038/sj.onc.1204815 11641782

[21] Kim K Kwon O Ryu TY Jung CR Kim J Min JK. Propionate of a microbiota metabolite induces cell apoptosis and cell cycle arrest in lung cancer. Mol Med Rep. (2019) 20:1569–74. doi: 10.3892/mmr.2019.10431 PMC6625448 31257531

[22] Cao Y Arbiser J D’Amato RJ D’Amore PA Ingber DE Kerbel R. Forty-year journey of angiogenesis translational research. Sci Transl Med. (2011) 3:114rv3. doi: 10.1126/scitranslmed.3003149 PMC8265598 22190240

[23] Yu DC Waby JS Chirakkal H Staton CA Corfe BM. Butyrate suppresses expression of neuropilin I in colorectal cell lines through inhibition of Sp1 transactivation. Mol Cancer. (2010) 9:276. doi: 10.1186/1476-4598-9-276 20950431 PMC2974727

[24] Sawa H Murakami H Ohshima Y Murakami M Yamazaki I Tamura Y. Histone deacetylase inhibitors such as sodium butyrate and trichostatin A inhibit vascular endothelial growth factor (VEGF) secretion from human glioblastoma cells. Brain Tumor Pathol. (2002) 19:77–81. doi: 10.1007/BF02478931 12622137

[25] Zhao Y You W Zheng J Chi Y Tang W Du R. Valproic acid inhibits the angiogenic potential of cervical cancer cells via HIF-1α/VEGF signals. Clin Transl Oncol. (2016) 18:1123–30. doi: 10.1007/s12094-016-1494-0 26942921

[26] Yang J Antin P Berx G Blanpain C Brabletz T Bronner M. Guidelines and definitions for research on epithelial-mesenchymal transition. Nat Rev Mol Cell Biol. (2020) 21:341–52. doi: 10.1038/s41580-020-0237-9 PMC7250738 32300252

[27] Lamouille S Xu J Derynck R. Molecular mechanisms of epithelial-mesenchymal transition. Nat Rev Mol Cell Biol. (2014) 15:178–96. doi: 10.1038/nrm3758 PMC4240281 24556840

[28] Lan X Lu G Yuan C Mao S Jiang W Chen Y. Valproic acid (VPA) inhibits the epithelial-mesenchymal transition in prostate carcinoma via the dual suppression of SMAD4. J Cancer Res Clin Oncol. (2016) 142:177–85. doi: 10.1007/s00432-015-2020-4 PMC11819227 26206483

[29] Mao S Lu G Lan X Yuan C Jiang W Chen Y. Valproic acid inhibits epithelial−mesenchymal transition in renal cell carcinoma by decreasing SMAD4 expression. Mol Med Rep. (2017) 16:6190–9. doi: 10.3892/mmr.2017.7394 28901475

[30] Taipale J Chen JK Cooper MK Wang B Mann RK Milenkovic L. Effects of oncogenic mutations in Smoothened and Patched can be reversed by cyclopamine. Nature. (2000) 406:1005–9. doi: 10.1038/35023008 10984056

[31] Phillips RJ Mestas J Gharaee-Kermani M Burdick MD Sica A Belperio JA. Epidermal growth factor and hypoxia-induced expression of CXC chemokine receptor 4 on non-small cell lung cancer cells is regulated by the phosphatidylinositol 3-kinase/PTEN/AKT/mammalian target of rapamycin signaling pathway and activation of hypoxia inducible factor-1alpha. J Biol Chem. (2005) 280:22473–81. doi: 10.1074/jbc.M500963200 15802268

[32] Tremaroli V Bäckhed F. Functional interactions between the gut microbiota and host metabolism. Nature. (2012) 489:242–9. doi: 10.1038/nature11552 22972297

[33] Singh N Gurav A Sivaprakasam S Brady E Padia R Shi H. Activation of Gpr109a, receptor for niacin and the commensal metabolite butyrate, suppresses colonic inflammation and carcinogenesis. Immunity. (2014) 40:128–39. doi: 10.1016/j.immuni.2013.12.007 PMC4305274 24412617

[34] Salem HA Wadie W. Effect of niacin on inflammation and angiogenesis in a murine model of ulcerative colitis. Sci Rep. (2017) 7:7139. doi: 10.1038/s41598-017-07280-y 28769047 PMC5541000

[35] Yonezawa T Kobayashi Y Obara Y. Short-chain fatty acids induce acute phosphorylation of the p38 mitogen-activated protein kinase/heat shock protein 27 pathway via GPR43 in the MCF-7 human breast cancer cell line. Cell Signal. (2007) 19:185–93. doi: 10.1016/j.cellsig.2006.06.004 16887331

[36] Elangovan S Pathania R Ramachandran S Ananth S Padia RN Lan L. The niacin/butyrate receptor GPR109A suppresses mammary tumorigenesis by inhibiting cell survival. Cancer Res. (2014) 74:1166–78. doi: 10.1158/0008-5472.CAN-13-1451 PMC3944627 24371223

[37] Lachner M O’Carroll D Rea S Mechtler K Jenuwein T. Methylation of histone H3 lysine 9 creates a binding site for HP1 proteins. Nature. (2001) 410:116–20. doi: 10.1038/35065132 11242053

[38] Zegerman P Canas B Pappin D Kouzarides T. Histone H3 lysine 4 methylation disrupts binding of nucleosome remodeling and deacetylase (NuRD) repressor complex. J Biol Chem. (2002) 277:11621–4. doi: 10.1074/jbc.C200045200 11850414

[39] Seto E Yoshida M. Erasers of histone acetylation: the histone deacetylase enzymes. Cold Spring Harb Perspect Biol. (2014) 6:a018713. doi: 10.1101/cshperspect.a018713 24691964 PMC3970420

[40] Ramaiah MJ Tangutur AD Manyam RR. Epigenetic modulation and understanding of HDAC inhibitors in cancer therapy. Life Sci. (2021) 277:119504. doi: 10.1016/j.lfs.2021.119504 33872660

[41] Das CM Aguilera D Vasquez H Prasad P Zhang M Wolff JE. Valproic acid induces p21 and topoisomerase-II (alpha/beta) expression and synergistically enhances etoposide cytotoxicity in human glioblastoma cell lines. J Neurooncol. (2007) 85:159–70. doi: 10.1007/s11060-007-9402-7 17534580

[42] Hatayama H Iwashita J Kuwajima A Abe T. The short chain fatty acid, butyrate, stimulates MUC2 mucin production in the human colon cancer cell line, LS174T. Biochem Biophys Res Commun. (2007) 356:599–603. doi: 10.1016/j.bbrc.2007.03.025 17374366

[43] Li Q Ding C Meng T Lu W Liu W Hao H. Butyrate suppresses motility of colorectal cancer cells via deactivating Akt/ERK signaling in histone deacetylase dependent manner. J Pharmacol Sci. (2017) 135:148–55. doi: 10.1016/j.jphs.2017.11.004 29233468

[44] Uribe-Herranz M Rafail S Beghi S Gil-de-Gómez L Verginadis I Bittinger K. Gut microbiota modulate dendritic cell antigen presentation and radiotherapy-induced antitumor immune response. J Clin Invest. (2020) 130:466–79. doi: 10.1172/JCI124332 PMC6934221 31815742

[45] Xiao X Cao Y Chen H. Profiling and characterization of microRNAs responding to sodium butyrate treatment in A549 cells. J Cell Biochem. (2018) 119:3563–73. doi: 10.1002/jcb.v119.4 29231270

[46] Gomes S Baltazar F Silva E Preto A. Microbiota-derived short-chain fatty acids: new road in colorectal cancer therapy. Pharmaceutics. (2022) 14:2359. doi: 10.3390/pharmaceutics14112359 36365177 PMC9698921

[47] Zagato E Pozzi C Bertocchi A Schioppa T Saccheri F Guglietta S. Endogenous murine microbiota member Faecalibaculum rodentium and its human homologue protect from intestinal tumour growth. Nat Microbiol. (2020) 5:511–24. doi: 10.1038/s41564-019-0649-5 PMC7048616 31988379

[48] Zhou Y Ji X Chen J Fu Y Huang J Guo R. Short-chain fatty acid butyrate: A novel shield against chronic gastric ulcer. Exp Ther Med. (2021) 21:329. doi: 10.3892/etm.2021.9760 33732302 PMC7903393

[49] Okabe S Okamoto T Zhao CM Chen D Matsui H. Acetic acid induces cell death: an in *vitro* study using normal rat gastric mucosal cell line and rat and human gastric cancer and mesothelioma cell lines. J Gastroenterol Hepatol. (2014) 29 Suppl 4:65–9. doi: 10.1111/jgh.2014.29.issue-s4 25521736

[50] Yan J Xu YH. Tributyrin inhibits human gastric cancer SGC-7901 cell growth by inducing apoptosis and DNA synthesis arrest. World J Gastroenterol. (2003) 9:660–4. doi: 10.3748/wjg.v9.i4.660 PMC4611423 12679905

[51] Kim YL Lee W Chung SH Yu BM Lee YC Hong J. Metabolic alterations of short-chain fatty acids and TCA cycle intermediates in human plasma from patients with gastric cancer. Life Sci. (2022) 309:121010. doi: 10.1016/j.lfs.2022.121010 36181864

[52] Schoeller A Karki K Jayaraman A Chapkin RS Safe S. Short chain fatty acids exhibit selective estrogen receptor downregulator (SERD) activity in breast cancer. Am J Cancer Res. (2022) 12:3422–36. PMC9360213 35968335

[53] Coutzac C Jouniaux JM Paci A Schmidt J Mallardo D Seck A. Systemic short chain fatty acids limit antitumor effect of CTLA-4 blockade in hosts with cancer. Nat Commun. (2020) 11:2168. doi: 10.1038/s41467-020-16079-x 32358520 PMC7195489

[54] Hu C Xu B Wang X Wan WH Lu J Kong D. Gut microbiota-derived short-chain fatty acids regulate group 3 innate lymphoid cells in HCC. Hepatology. (2023) 77:48–64. doi: 10.1002/hep.32449 35262957 PMC9970019

[55] Lucas S Omata Y Hofmann J Böttcher M Iljazovic A Sarter K. Short-chain fatty acids regulate systemic bone mass and protect from pathological bone loss. Nat Commun. (2018) 9:55. doi: 10.1038/s41467-017-02490-4 29302038 PMC5754356

[56] Luu M Riester Z Baldrich A Reichardt N Yuille S Busetti A. Microbial short-chain fatty acids modulate CD8(+) T cell responses and improve adoptive immunotherapy for cancer. Nat Commun. (2021) 12:4077. doi: 10.1038/s41467-021-24331-1 34210970 PMC8249424

